# Exploration of psychological distress experienced by survivors of adolescent cancer reporting a need for psychological support

**DOI:** 10.1371/journal.pone.0195899

**Published:** 2018-04-17

**Authors:** Malin Ander, Jenny Thorsell Cederberg, Louise von Essen, Emma Hovén

**Affiliations:** 1 Clinical Psychology in Healthcare, Department of Women’s and Children’s Health, Uppsala University, Uppsala, Sweden; 2 Pediatric Oncology, Department of Women’s and Children’s Health, Uppsala University, Uppsala, Sweden; Monash University, Institute of Cognitive and Clinical Neurosciences, AUSTRALIA

## Abstract

**Objective:**

In this qualitative study, we aimed to provide an in-depth exploration of cancer-related psychological distress experienced by young survivors of cancer during adolescence reporting a need for psychological support.

**Methods:**

Two individual interviews were held with ten young survivors of cancer diagnosed in adolescence. The interviews were audio-recorded and transcribed verbatim. Analysis followed the guidelines for inductive qualitative manifest content analysis.

**Results:**

The survivors described distress experienced during and after the end of treatment. Five categories comprising 14 subcategories were generated. The categories included: A tough treatment, Marked and hindered, Not feeling good enough, Struggling with the fragility of life, and finally, An ongoing battle with emotions.

**Conclusion:**

Young survivors of adolescent cancer reporting a need for psychological support described feeling physically, socially, and mentally marked by the cancer experience. They struggled with powerlessness, insecurity, social disconnection, loneliness, and feelings of being unimportant and a failure, and had difficulties understanding and managing their experiences. These concerns should be addressed in psychological treatments for the population irrespective of which approach or model is used to understand survivors’ difficulties. A trans-diagnostic approach targeting processes that underpin different manifestations of distress may be effective.

## Introduction

A cancer diagnosis during adolescence infers physical, emotional, existential, and social cancer-related stressors while at the same time managing developmental changes and experiences associated with preparation for adulthood [1]. The World Health Organization [2] defines adolescence as the period between 10 and 19 years, however, other age ranges have been used to define adolescence in psycho-oncological research. From a psychological perspective, adolescence is considered a critical period characterised by key developmental challenges including movement toward independence, development of identity and relationship skills, and increased awareness of morals and values [3]. The first onset of several mental disorders typically occurs during adolescence [4], social contexts and peer and parent interactions appear to play significant roles in the development of psychological morbidity during this period [5]. Given this combination of stress exposure and developmental vulnerability, there is a need to pay attention to the needs of persons diagnosed with cancer during adolescence [6].

Findings are inconclusive with regard to distress experienced by persons diagnosed with cancer during adolescence in comparison to controls [7–9]. A subgroup reporting clinically relevant levels of symptoms of anxiety, depression, and/or posttraumatic stress can be identified across studies, and some develop symptoms years after diagnosis and completion of treatment [10]. Moreover, a subgroup reports an unmet need of psychological support following treatment completion [11, 12]. Empirically supported psychological treatments to address this need are lacking. The majority of studies investigating psychological distress after treatment completion among young persons diagnosed with cancer during adolescence have investigated the occurrence of symptoms of depression, anxiety, and/or posttraumatic stress as outlined in psychiatric classification systems such as in the Diagnostic and Statistical Manual of Mental Disorders (DSM) [13] and used self-reports to measure the distress. However, investigated symptoms may not adequately capture the phenomenon of distress experienced by survivors of adolescent cancer.

The role of cognitive behavioural therapy (CBT) to address disease-specific psychological distress has received increased attention. The Medical Research Council (MRC) guidance for developing effective, sustainable, and implementable complex interventions, underscores the importance of thorough development-, feasibility-, and pilot work to prepare for successful implementation at an early stage [14]. The present study represents phase I development work, aiming to identify theory and contribute to a thorough understanding of the problems to be addressed in an intervention for the population.

Some studies have explored long-term experiences related to cancer diagnosed during adolescence. Identified experiences include, e.g., appearance-related concerns [15], unmet physical, psychological and social needs [16], and managing cancer-related fertility matters [17]. Not only challenges, but also positive consequences have been identified [18]. To the best of our knowledge, there is a lack of studies that specifically explore psychological distress experienced by young survivors of cancer diagnosed during adolescence reporting a need for psychological treatment. Such knowledge is crucial to the development of effective psychological interventions for the population.

The aim was to explore cancer-related psychological distress experienced by young survivors of cancer during adolescence reporting a need for psychological treatment. In this study, distress refers to an unpleasant experience of a psychological, i.e., cognitive, behavioural, emotional, social, or spiritual nature [19, 20].

## Materials and methods

This report follows the COREQ checklist for reporting qualitative research [21].

The study was conducted as part of an open trial in which we developed a cognitive behavioural conceptualisation of psychological distress related to adolescent cancer and tested the feasibility of individualised face-to-face CBT for this distress (trial ID ISRCTN15975376), of which the primary outcomes have not yet been reported. The original trial protocol is available in S1 Trial Protocol.

### Design and sample

The study had an explorative design. Eligibility included being 15–25 years at study-start, having completed cancer treatment for a cancer diagnosis during adolescence (13–19 years) reporting a need for psychotherapy at study-start, being able to participate in individual face-to-face CBT once a week up to 15 weeks. Exclusion criteria included ongoing psychotherapy and/or displaying severe psychiatric symptoms in need of treatment based on a clinical assessment combining information from the Montgomery-Åsberg Depression Rating Scale-Self report (MADRS-S) [22] and the MINI neuropsychiatric interview [23].

Persons treated at the pediatric oncology center in Stockholm or Uppsala in Sweden were identified via the Swedish Childhood Cancer Registry (n = 234). Telephone numbers to potential participants 18 years of age or older were searched for via internet search engines. For potential participants 15–17 years of age, telephone numbers to those who had completed cancer treatment (according to medical records) were retrieved via the pediatric oncology centers (n = 60). A total of 201 potential participants were invited to participate either via telephone (n = 90), via parents who received brief information via telephone (n = 14) or via postal letter (n = 97). Reasons for not being invited to participate were: incomplete treatment (n = 17), declining information via telephone (n = 4), impossibility of identifying a correct number or address (n = 3), moves abroad/excessive distances away for participation (n = 3), administrative failure (n = 3), deceased during inclusion (n = 1), turned 26 during inclusion (n = 1), and prior contact with study therapists (n = 1). Participants who were invited via telephone and declined participation (n = 81) were asked to provide reasons for not wanting to participate. The most common reason was not having a need for psychological support (n = 61, 68%). Other reasons include current psychotherapy (n = 7), too long travel distance to study sites and/or time constraints (n = 6); interested but not ready to commit to the study (n = 5), and not wanting psychotherapy (n = 2).

Persons who expressed interest in participating (n = 13) were invited to an eligibility interview. One person did not complete the interview and declined participation because of travel distance. Another person was excluded due to ongoing psychotherapy. Eleven participants were invited to participate. One person considered participation too demanding and decided not to participate. For the only person aged 15–17 years, an assessment of whether the person had sufficient understanding of the project and realized what participation implied was carried out to examine whether parental consent was needed, which it was not and parental consent was thus not obtained. This procedure was approved by the research ethics committee. Ten participants ultimately took part. Participants’ clinical and demographic characteristics are presented in Table 1.

**Table 1:** Participant characteristics.

	*M*	*SD*	*Range*
Age at study start (years)	21.0	2.8	17–25
Age at diagnosis (years)	15.9	1.4	13–17
Time since self-reported end of treatment (years)	4.2	2.2	1–8
MADRS-S^1^ BAI^2^ PCL-C^3^	16.7 14.3 37.2	5.9 15.5 13.4	8–27 1–56 20–70
	*N*		
Female/Male	4/6		
Type of cancer			
Leukemia	4		
CNS tumor	2		
Lymphoma	2		
Soft tissue sarcoma	1		
Other malignancy	1		
History of relapse	1		
Living situation			
With parents	7		
Alone or with partner/friend	3		
Relationship status			
Single	5		
In a relationship	5		
Employment status			
Student	6		
Employed full-time	1		
Employed part-time	2		
Unemployed	1		
Part-time sick leave	3		
Self-reported late effects yes^4^/no	8/2		

^1^ Montgomery Åsberg Depression Scale- Self report, total score range 0–54

^2^ Beck Anxiety Inventory, total score range 0–63

^3^ PTSD Checklist–Civilian Version, total score range 17–85.

^4^ Self-reported late effects included alopecia, anemia, balance problems, fatigue/tiredness, headaches, high blood pressure, loss of appetite, kidney stone, memory problems, nausea, nerve pain, speech difficulties, sudden numbness, thrombus, visual impairment.

Six participants had received psychotherapy or counseling. Of these, five had received psychotherapy or counseling after the end of cancer treatment. Two of the participants who had previously received psychotherapy had received CBT for obsessive-compulsive disorder and two for depression. Two had received psychotherapy or counseling from social workers or psychologists with unknown or unclear therapeutic approach. These treatments had addressed worry, panic, depression, drug abuse, and/or aggression. Four reported that they had been prescribed medication for psychological distress. Time since the end of psychotherapy or counseling ranged from 1 to 7 years (*m* = 3.5 years).

Inclusion continued until all individuals who had been identified via the Swedish Cancer Registry had been invited and screened for eligibility.

### Data collection

Data on age and date of diagnosis and type of cancer were retrieved from the Swedish Childhood Cancer Registry. Following inclusion, participants were asked about: demographics, clinical variables (e.g., recurrence, time since the end of their treatment, their type of treatment, their late effects), and previous receipt of psychotherapy or counseling. Moreover, a number of self-report questionnaires measuring psychological variables were administered of which symptoms of depression [22], anxiety (Beck Anxiety Inventory, BAI [24]), and posttraumatic stress (PTSD Checklist- Civilian Version, PCL-C [25]) are presented in the present study. Due to administrative failure, one participant answered the self-reported questionnaires ten weeks after the planned date (after six sessions of CBT).

Following inclusion and completion of questionnaires, participants were interviewed twice within two weeks. Three clinical psychologists trained in CBT, two women and one man, conducted the interviews (the first author, MA, was one of the interviewers). At the time of the study, all worked in our research group, two as PhD students and one as a post doc researcher. All had previous experience of collecting and/or analysing qualitative research data. Participants were interviewed by the same interviewer on both occasions. To improve the quality of the interviews, at least one of the first interviews from each interviewer was reviewed by a senior researcher with extensive experience in qualitative interviewing.

The interviews were performed in two settings. In Uppsala, in one of two rooms located at the pediatric oncology ward where participants had received treatment. In Stockholm, interviews were conducted in a private psychology practice. Before the start of the interview, participants were informed about the purpose of the interview, of how their answers should be handled, of the plan for the interview to last for about an hour, of the parameter that participants were to decide for themselves what they wanted to share, of the parameter that they could stop the interview whenever they wanted without providing a reason and the parameter that pauses could be included if needed. All of the interviews were audio-recorded.

All interviews started with the question: Please tell me how you think and feel about having had cancer. Probes like “*Tell me more about*…”, “*Tell me how you felt when…”*, *and Please tell me what you mean by…*” were used to encourage elaboration, reflection, and clarification. The first interview with each participant was listened to carefully by one or two of the interviewers to identify areas that would benefit from further exploration in the second interview with the same participant. Interviews lasted from 33 to 104 minutes. Two interviews included a pause.

### Data analysis

Analysis followed the guidelines for inductive qualitative manifest content analysis outlined by Graneheim and Lundman [26]. Audio-recorded interviews were transcribed verbatim by a professional transcription agency. The first author (MA) carefully read all transcripts and listened to all audio-recordings to detect any mistakes in the transcriptions and to get a sense of the entirety of the material. MA identified and condensed meaning units relating to unpleasant experiences of a psychological, i.e., cognitive, behavioural, emotional, social, or spiritual nature. The second author (JTC) listened to all audio-recordings, read all transcripts, and reviewed and condensed meaning units. Following a discussion between MA and JTC, a number of meaning units were added and a few were removed. The analysis was thereafter conducted in an iterative abstraction process as follows: i) condensed meaning units were coded by MA and JTC independently, and codes were then compared, discussed, and revised if needed; ii) MA and JTC independently scrutinised codes based on differences and similarities and sorted them into preliminary higher order categories and subcategories which were compared, discussed, and further elaborated; and iii) MA, JTC, and the last author (EH) reviewed the condensed meaning units, codes, subcategories, and categories individually with respect to whether they were grounded in data. Coherence in levels of abstraction, congruence between and within all levels of abstraction (including attention to deviant cases), and whether the categories and the labels satisfactorily reflected psychological distress [27] and covered the data [26] were also reviewed. Individual reviewing was followed by joint discussions regarding disagreements, incongruences, and ambiguities which guided further refinement of the labelling and grouping of codes, subcategories, and categories. This process was repeated until MA, JTC, and EH considered all data to be accurately covered and reflected by identified categories.

MA, JTC, and EH kept reflexive logs of methodological and theoretical ideas and assumptions and the potential influence of their theoretical and experiential backgrounds on the data analysis throughout the analysis.

### Ethics

The study was approved by the Regional Ethical Review Board in Uppsala, Sweden (2014/443). All participants were provided written and oral information about the study, which included that participation was voluntary, that it was possible to withdraw without any consequences at any time, and the precautions that would be taken to protect data confidentiality. Informed consent was obtained from all participants. All data were handled according to Swedish legislation, *Personuppgiftslagen* (PUL 1998:204) and *Patientdatalagen* (2008:355). To ensure confidentiality, quotes are presented only with gender and age range.

## Results

The data were sorted in: distress experienced during vs after end of treatment.

Five categories comprising 14 subcategories were generated, see Table 2. One category covered descriptions of distress experienced during treatment and four categories covered distress experienced after the end of treatment.

**Table 2:** Categories and subcategories of distress reported by young survivors of adolescent cancer reporting a need for psychological support.

	Categories	Subcategories	Meaning units
During treatment	A tough treatment	A mental challenge	“But in the middle of this fatigue, I had anxiety … I felt like shit. So I just buried all my feelings and just ‘was’. I, like, couldn’t do anything so … I just wanted to turn off my thoughts, but that was … the reason I couldn’t sleep at night because I couldn’t ever wind down, because I had so many thoughts in my head.” (ID 7, Woman, 20–25 yrs., 15–17 yrs. at diagnosis)
		Particularly stressful events	“So when … when the nurse said to me, yes, you have leukemia, I thought … it didn’t sink in … it didn’t hit me until I … they started the treatment after … it didn’t even hit me then … I think it hit me a week after they started treating me.” (ID 10, Man 20–25 yrs., 15–17 yrs. at diagnosis)
		Loss of control over one’s life and body	“But this is probably the most pronounced … in everything, that you just have to live through the period knowing that you might not make it. Because even if you don’t admit it to anyone, that is absolutely the hardest thing about cancer. You know, the fear … the thing that sticks out the most, I mean … the fear of dying.” (ID 4, Man 20–25 yrs., 15–17 yrs. at diagnosis)
		Lonely and disconnected	“All of my best friends that I’d been with since preschool stopped getting in touch … and … when I came home from treatment and all that, when I was out walking, they could walk past me without even saying anything.” (ID 7, Woman, 20–25 yrs., 15–17 yrs. at diagnosis)
After the end of treatment	Marked and hindered	Cancer free but not free	“You feel like … like old and… sick (…) Well, not old, but like, it doesn’t feel like … this is how I should feel … or … someone should do in… when you’re, like, XX years old.” (ID 9, Man, 15–19 yrs., 15–17 yrs. at diagnosis)
		Being the one with cancer	“Either I have to, like, say no, it’s fine … you … all … I’m fine now, or else they have to … if it’s just hanging there in the air all the time when you meet someone and it’s also really hard that you … feel like you have to clear the air all the time, when you see people. Which means that you’d almost rather not bother talking to anyone, who you don’t know, I mean … who you’re not close with … it’s really tiresome having to keep bringing it up all the time, I think.” (ID 4, Man 20–25 yrs., 15–17 yrs. at diagnosis)
	Not feeling good enough	Not being in the right place in life	“Maybe I shouldn’t have let myself just get dragged down and just shut down, or, I don’t know. Maybe I should have tried to keep up my social life more or something or like … It-It’s more that it feels like that … I let things fall away and, like, shut down.” (ID 3, Woman, 20–25 yrs., 15–17 yrs. at diagnosis)
		Insecure and left out	“I’ve had kind of a hard time getting into that part, the social part, after this. I think … I’ve, like, gotten used to talking with so many older people that I’ve had kind of a hard time sometimes being in the young world and I’m a little more used to them … the adult world in some way, which means it’s a little hard for me to adapt in social settings with them sometimes.” (ID 1, Man, 15–17 yrs., 13–15 yrs. at diagnosis)
	Struggling with the fragility of life	Affected by people’s withdrawal	“But that … you always go around worried that this, but … if-if something, like, too ‘different’ happens then like … then you’re on your own anyway. It’s, like, a really uncomfortable feeling, I think.” (ID 5, Man, 20–25 yrs., 15–17 yrs. at diagnosis)
		Everything may soon be gone	“… but it’s like, you know … if I were to get it back and know that I’d done my last three years at school … and like couldn’t afford anything other than eating noodles, to take it to the extreme [short laugh]. Well … then, yeah. That’s what’s, like, there in the background somewhere, that you want to … that you want to, like, live qualitatively, because something might happen.” (ID 4, Man 20–25 yrs., 15–17 yrs. at diagnosis)
		Lost time and lost hope	“I don’t develop and I don’t have a job and I don’t have any friends and I, like, don’t have anything.” (ID 8, Woman, 15–19 yrs., 13–15 yrs. at diagnosis)
	An ongoing battle with emotions	Not understanding own feelings	“… then I think, man, compared to how it was, I have it pretty freaking good now and so I should … like, feel good. But I’m still, like …pretty down a lot and stuff. It’s kind of hard to understand, you know, that I … why I’m like this now. I mean, I understand why it was like that then but … why it’s still like this.” (ID 5, Man, 20–25 yrs., 15–17 yrs. at diagnosis)
		Drained by feelings	“… like if I … take it easy or do … nothing, like, I just … but if I’m home by myself and just … am going to watch a movie or something like that, then I can get one of those … some type of … stress feeling like, man, now it’s just like it was then, now I’ve got to do something or something like that. So it’s kind of hard to … (…) It’s, like, difficult for me to just lie in bed and take it easy … um … and like that, because I get … I become … it, like, stresses me out. There are several reasons for it, too. It’s because … that was all I did then and that feeling is like really, it’s really associated with … with, like, how I felt then.” (ID 5, Man, 20–25 yrs., 15–17 yrs. at diagnosis)
		Fighting one’s feelings	“Then it’s really weird and really tough because I don’t … I can’t, like, handle anything that has anything at all to do with cancer.” (ID 5, Man, 20–25 yrs., 15–17 yrs. at diagnosis)

### A tough treatment

#### A mental challenge

The treatment period was described as tough and mentally demanding. Participants described having difficulties remembering what had happened, and what they had thought and felt. Memories of the treatment were sometimes described as a mess or an empty hole. All that the participant had wanted was for the treatment to be over and that their lives would return to ‘normal’. Some had passively waited for time to pass, while others had struggled to maintain a sense of normality, e.g., by trying to keep up with school and attending parties despite being very sick. Emotional numbness and helpless passivity alongside active efforts to detach from the body, the seriousness of the situation, fears, sadness due to losses, and other painful experiences were described. Some expressed that they had felt as if they were observing themselves from outside, and interpreted this as a way to distance themselves from what they were going through.

#### Particularly stressful events

Receiving the diagnosis, being informed about treatment changes or prolongation, experiencing dramatic treatment complications (for example, waking up unable to move or talk after life-threatening infections), losing one’s hair, receiving radiation, and strong pain due to treatment side effects were mentioned as particularly stressful. Participants described that they had reacted to these experiences with anger, disappointment, emotional emptiness, grief, hopelessness, panic, powerlessness, shock, uncertainty, feelings of unfairness, confusion, and de-realisation. Having become scared and anxious by seeing parents cry or acting concerned was also mentioned.

#### Loss of control over one’s life and body

Participants described that they had struggled with losing control over their lives and bodies, and had felt powerless and dependent during treatment. Thoughts such as “Will I survive?”, “What is going to happen?”, and “Will I make it?” were described and conceptualised as fear of dying. Some reported a diminishing fear while others described a recurrent fear of dying during treatment.

Participants described that they had felt forced to give up all control over their bodies to experts who knew more about their bodies than they did themselves. This had caused a feeling of estrangement, as if the sick body was not theirs, and of not being able to trust their bodies. Becoming very dependent on others and needing help with absolutely everything, including one’s hygiene, was described as traumatic and humiliating. Participants had felt that others knew too much about them, which had made them feel frustrated, exposed, and sometimes offended. Refusing a medical procedure and occasionally not taking their medication were described as strategies to regain a sense of control over their bodies. Further, obsessive-compulsive behaviours related to, e.g., symmetry and cleaning were mentioned as strategies used to regain control.

#### Lonely and disconnected

Participants described that the disease had caused a loss of social context and that they had felt stuck while peers’ lives had moved forward without problems. This had triggered frustration, feelings of injustice, and a sense of missing out. Having felt abandoned by friends and having been avoided by people in school and public spaces were further mentioned. This had made some give up social efforts, avoid school, and thus increase isolation. Further, not feeling allowed, or daring, to talk about one’s fears and worries, including the fear of dying, and consequently, feeling very lonely with one’s fears and worries was expressed.

### Marked and hindered

#### Cancer free but not free

Participants described the disease as a handicap and expressed frustration and grief by not being able to escape or erase its lifelong impact. Feeling constantly drawn back by new health problems was mentioned and participants expressed that it was unfair that they had to feel sick and worry about their health when they were so young.

An underlying fear of recurrence was manifested as worry in response to disease symptoms, especially symptoms resembling those experienced before the cancer diagnosis. Participants expressed a fear of missing critical symptoms, difficulties interpreting physical symptoms, and not trusting their bodies. Checking and analysing their bodies, seeking reassurance from others, using positive self-talk, and searching the internet for information were used as strategies to calm themselves, however they sometimes were perceived as worsening the fear. Fear of the mental consequences of falling ill again was furthermore described, with doubts about whether they would manage to go through it all once more.

Moreover, participants struggled with poor concentration, learning difficulties, fatigue, and speech difficulties, which negatively affected their social functioning. They expressed dissatisfaction with their bodies and appearance, missed their pre-cancer looks and worried about their weight and body shape. Changes in appearance were constant reminders of the disease and some exercised hard to regain their former appearance

#### Being the one with cancer

Participants described being treated differently. They felt that they had lost their identity and that people saw them as ‘the one with cancer’. To avoid this, some did not talk about cancer-related experiences with friends and some did not mention the cancer to people who did not know about it. However, ‘hiding’ the experience sometimes triggered anxiety due to feeling dishonest. Participants further described feeling pitied and victimised by others who, e.g., avoided them or became scared and hesitant in their company. This caused anger and feelings of uneasiness and inferiority. Some felt responsible for and tried to alleviate others’ distress by assuring them that everything was fine, irrespective of how they felt, and sometimes avoided certain people or situations because they felt tired of handling others’ reactions.

Others’ expectations about positive cancer-related consequences, e.g., ‘having learned so much from cancer’, caused frustration and made participants feel different and judged. Still, mixed feelings were described. While not wanting to be treated differently or pitied, they wanted people to understand the ongoing impact of the disease.

For some, the illness had caused fundamental changes in relationships with family members. Participants described that they felt babied, overprotected, controlled as well as not listened to or understood by parents, which led to frustration and feelings of dependence. Experiencing that it was uncomfortable and difficult to share difficult cancer-related experiences with loved ones and hiding distressing thoughts and feelings from family members, not being more of a burden than one already was or had been, were described.

### Not feeling good enough

#### Not being in the right place in life

Participants expressed feelings of worthlessness and failure in relation to where they were in life, what they had accomplished, and how they had handled their struggles. They described that their “normal” teenage development had been ruined by the cancer, that they were lagging behind everybody else socially, and that they were held back and stuck. Some described feeling younger and more dependent than peers, others described feeling older and more mature, and some described mixed feelings.

When treatment was completed, some expressed having experienced stress and frustration in relation to returning to school and social life while at the same time having to recover mentally and physically from the disease. Regret and thinking that they should have been more active and put more effort into school and social life when they were sick were mentioned. Along with this, participants expressed feelings of stress, guilt, and anxiety about not living life to the fullest and not making the most of time. Some felt incapable of handling their lives, and were frustrated about not being able to break away from their own futile patterns.

#### Insecure and left out

Insecurity and lack of confidence, especially with regard to social performance was described. Not knowing who they are and what they want and like as well as doubting their ability to perform, e.g., at work was expressed, which often led to procrastination. Participants further described being vulnerable to others’ opinions and feeling introverted and suspicious towards others. They were anxious in social settings and some described that they withdrew socially or tried very hard to adapt to others needs’ or wishes because of fear of degradation and rejection.

Frustration, anger, and sadness for being overlooked, misunderstood, and unappreciated were described. A sense of being different from everybody else was expressed in relation to a wish ‘to be normal’. Some expressed discontent with their social lives and longed for more meaningful relationships.

### Struggling with the fragility of life

#### Affected by people’s withdrawal

Participants described lingering disappointment, sadness, and frustration about how people in school had treated them when they were sick. When they had gone through the toughest time of their lives and had been more vulnerable than ever, persons they had thought of as friends had abandoned them and had not seemed to care. Participants had difficulties understanding these actions and feared being let down again. Wanting to confront people who had let them down, feeling a loss of trust in friends and a feeling of only being accepted if fulfilling a certain social role were described.

#### Everything may soon be gone

Participants described thinking about mortality and a need to understand why they got cancer. The experience had left them feeling that you can lose everything in one moment. On the one hand, this made them value their relationships more and not stress about unimportant things, on the other hand it made it difficult for them to pursue long-term goals. Some expressed feeling unimportant, thinking that even if their own lives would end, others’ would go on and people would get over or even forget about them.

#### Lost time and lost hope

Participants described a feeling of having lost everything that was important and that the cancer had ruined their lives and their chances to get the future they wanted. Some described that they missed and grieved over their previous lives and former selves, and felt deprived of their teenage years. Meaninglessness and hopelessness regarding the ability to cope with common life adversities and loss of motivation to try to achieve things and move forward in life were described along with thoughts that it would have been better if they had died from cancer.

### An ongoing battle with emotions

#### Not understanding one’s own feelings

Participants described how they dismissed and did not understand their reactions and feelings. They sometimes had difficulties identifying, articulating, and trusting them, let alone difficulty knowing how to act on them. In some situations, they expected to feel happy or relieved, e.g., when treatment was completed or when everything was fine at follow-ups, but instead they felt sad or depressed. Fear of using the cancer experience as an excuse to feel depressed was mentioned. Moreover, feelings of guilt, shame, and anger about not being happy and doubt about their right to feel the way they did were expressed.

#### Drained by feelings

Participants described struggles with anxiety and worry, recurrent panic attacks interspersed with feelings of numbness and hopelessness, obsessive thoughts, and fear of being judged. Triggers of anxiety such as thinking about the future, feeling pressured at work, being in public spaces, and feeling strong physical stress symptoms were described. Tension and difficulties relaxing were expressed.

Rigid distracting behaviours, compulsive behaviours, anticipatory worry, and brooding about the causes of feelings were described as strategies used to reduce anxiety. Some described these as efficient, while others described them as ineffective but unstoppable. Difficulties handling anxiety when alone and feeling overly dependent on loved ones for emotional support were expressed. Some described how their current problems had started during treatment. Others described their problems as present even before the diagnosis, but that the cancer had made them worse.

Participants described feelings of anger and injustice. One participant said she was acting out on every perceived injustice as a compensation for not having been able to stand up for herself when in treatment. However, outbursts of anger were often followed by feelings of guilt, regret, and sadness. Feelings of depression, numbness or emptiness, and difficulties feeling joy or pleasure were further described. Some expressed guilt, sadness, and frustration about having disrupted loved ones’ lives and having caused them suffering.

#### Fighting one’s feelings

Being reminded of cancer and thinking about it triggered anxiety, anger, and sadness and participants expressed ongoing struggles to keep away and reject painful cancer-related feelings e.g., by avoiding certain objects associated with treatment. Some had become used to shutting off distressing thoughts and feelings during treatment and described that they now were doing so automatically. They tried to ignore and distract themselves from their experiences by focusing on others’ problems, biting the bullet, and thinking positively. However, these strategies were described as mentally exhausting and unsustainable in the long-term.

Participants described that they had buried or blocked their thoughts and feelings, but that they could not ignore them any longer, and felt a need to talk about them even if they were very painful. However, they did not want other people to initiate these talks.

## Discussion

This study explored cancer-related psychological distress experienced by young survivors of cancer during adolescence reporting a need for psychological treatment. The survivors described distress experienced during and after the end of treatment. Previous studies have identified areas of distress similar to those identified in the present study, e.g., recurrent thoughts about the cancer experience and fear of relapse [18, 28, 29], feeling marked by the cancer experience [28], feeling left behind [29], and social isolation, alienation, and concerns about cancer disclosure [16, 18, 29, 30]. The present study adds to the existing knowledge by providing a more thorough description of salient concerns needed to inform development of engaging and effective psychological treatments.

A post-traumatic stress disorder model to understand adjustment to paediatric and adolescent cancer has been widely used, however the validity of this conceptualisation of cancer-related distress has been questioned [31]. Participants in our study described strong emotional reactions in response to events during treatment, e.g., being informed of the diagnosis or a recurrence, facing acute medical conditions, being abandoned by a friend, and losing hair. Not all described experiences would qualify as traumatic events according to the A-criterion in the DSM-V [13], which states that *“medical incidents that qualify as traumatic events involve sudden*, *catastrophic events”* ([13], p. 274). Knowledge about which stressors survivors of adolescent cancer perceive as challenging and about which reactions these cause is important to develop appropriate psychological interventions for the population. This includes identifying whether experiences are best understood using stress-related, anxiety, and/or depressive disorder frameworks.

In accordance with previous studies [18, 32], feelings of powerlessness and loss of control emerged as salient struggles during treatment. Powerlessness and helplessness were described in relation to the risk of dying, threatened integrity, and not being able to decide over the trajectory of one’s life. The experiences were linked to resentment, depression, anxiety, frustration, shame, and humiliation. Powerlessness is closely related to perceived control, and the role of perceived control, especially over anxiety-related events, has been emphasised in the etiology of anxiety disorders and suggested as a trans-diagnostic process that could be targeted in treatment. A person’s perceived control may or may not correspond with the person’s actual control [33]. When actual control is low, as with some cancer-related stressors, strategies aimed to adapt to the stressor may be more distress-reducing than strategies aimed to change the stressor [34]. Although participants expressed low perceived control over their health, body, interpersonal relations, and emotions during treatment, they had expected to regain control and power over their life after the completion of treatment. They had struggled to get through treatment using strategies such as pushing away thoughts and feelings, which could be classified as avoidance or disengagement coping [35]. Some of the strategies such as trying to think positively, distraction, and efforts to maintain one’s ‘normal’ life are often described as promoting adjustment to uncontrollable stressors [35], by offering a respite from stress and negative emotions. However, findings suggest that these strategies rather were used to avoid distressing thoughts and emotions and illustrates the importance of not only asking about the form and the self-reported aim of coping behaviours, but also help to explore their functions and consequences.

Participants had expected to feel safe and return to their pre-cancer life after treatment completion. Findings show that they instead experienced e.g., emptiness and stress, and that they continued to be faced with cancer-related uncertainty and stressors. The absence of expected relief and happiness and the unforeseen emerging stressors combined with limited energy due to lasting effects of disease and treatment led to frustration, stress, anxiety, resentment, and depression. These concerns may be important to address in treatment. Approaches such as behavioural activation (BA) [36] or Acceptance and Commitment Therapy (ACT) [37] could be useful to understand and treat depressive symptoms described by the survivors. Both underscore the importance of avoidance of aversive experiences as a factor in maintaining difficulties.

Loneliness and feeling disconnected from friends were identified as prominent aspects of distress during treatment. Participants had become socially isolated and alienated, either because others had withdrawn from them, or because they had withdrawn from others. In some cases, experiences of social exclusion and abandonment during treatment seemed to have had a lasting psychological impact on well-being, especially social relationships and self-esteem. Participants described a fear of being rejected and excluded if they deviated from what was expected, difficulties trusting others and feelings of being different, inferior, incompetent, and not accepted by others. Some of these experiences have been described by others [12, 18, 30]. Previous studies have not provided an in-depth exploration of social anxiety in young survivors of adolescent cancer. Such an exploration is warranted as social anxiety is the most common anxiety disorder in late adolescence and young adulthood [38], and as the cancer experience involved many stressors that may elicit social anxiety (e.g., social isolation, changed appearance). Following a cancer diagnosis, social exclusion and avoidance can be understood in terms of cancer stigma of which the primary cause is the fear of the disease itself and its association with death [39, 40]. As exemplified in this study, dealing with others’ exclusionary behaviours can increase social anxiety and lead to an increase of prosocial behaviours, e.g., becoming more compliant to the requests of others [41]. Although these actions may serve as important re-affiliative functions, they can also be problematic if the fear of exclusion becomes too strong and/or is persistently overestimated. Given experiences of exclusion and other negative social experiences during treatment, participants’ mistrust towards others is understandable. Cognitive behavioural models to understand social anxiety, typically posit that fear of evaluation, selective attention to cues of evaluation, maladaptive avoidance behaviours, and dysfunctional cognitions are the core factors involved in the maintenance of problems [42]. In the context of cancer, minority stress models may be relevant to fit with CBT models of social anxiety [43] to account for experiences related to stigma and alienation. Further, mistrust following experiences of perceived exclusion and betrayal was expressed and may be important to address in treatment.

Participants further described struggling with existential concerns. Yalom [44] outlined four key concerns considered essential to the human condition, namely death, freedom, isolation, and meaninglessness. The inevitability of death is seen by many as a fundamental source of human anxiety, and death anxiety is often intensified when one’s mortality is brought into focus, such as when one is faced with a serious illness. Awareness of one’s own death has been described as one of the loneliest experiences, closely connected with existential isolation, including the notion that we are all born and die alone [44]. In our study, the experience of feeling let down in an extremely vulnerable situation and feeling disconnected from one’s usual context while watching others’ lives go on were particularly salient. This elicited thoughts and feelings related to existential isolation and meaninglessness, e.g., a sense of being unimportant to others and to the world. Further, awareness of death’s inevitability and unpredictability elicited concerns with, e.g., daring to make long-term investments and how to live life to the fullest. Aspects of these latter concerns have been identified in previous studies [18]. With respect to understanding dysfunctional distress related to these concerns, models used to understand and reduce maladaptive worry, for example by targeting experiential avoidance or intolerance of uncertainty, may be useful [37, 45].

In the midst of struggles with social acceptance and inclusion, existential issues and regaining or building a new life, the survivors further struggled with confusion regarding who they were, what they liked, what they wanted, and what they longed for. Identity refers to our sense of who we are and identity development is one of the most important tasks of adolescence [46, 47]. Acknowledging the process of identity formation and supporting the exploration of values and knowledge about oneself has been put forth as important clinical considerations to promote engagement in CBT with youth [48] and may be particularly important to address in CBT with young cancer survivors.

The majority of research in the field of psychopathology has adopted a disorder-focused approach to develop effective treatments. However, the extensive comorbidity [49] and similarity between cognitive behavioural maintenance processes across disorders [50] alongside the disadvantages of using a categorical conceptualisation of psychological disorders have directed attention to trans-diagnostic perspectives on psychopathology. Trans-diagnostic approaches state that there are key maintenance processes (e.g., experiential avoidance and cognitive fusion [51]), that cut across psychological disorders [50]. Given the diverse manifestations of distress and the overlap of symptoms, a trans-diagnostic approach targeting processes that underpin different forms of cancer-related distress may be expedient and effective for young cancer survivors [52].

Some methodological aspects should be mentioned. The potential impact of interviewers and researchers on collection and analysis of data should be considered. We expect that the fact that participants were interviewed twice by licensed psychologists with research experience contributed to rich data. The trustworthiness of findings is supported by a systematic and rigorous, yet reflexive and iterative, data analysis. The interviewers and two of the researchers involved in the data analysis had clinical training in CBT which may have impacted how participants’ answers were interpreted and what probing questions they were asked in the interview. However, interviewers’ education in clinical psychology included extensive education in using open questions, active listening, and psychological processes involved in psychological suffering. This may have helped to detect and explore subtle nuances in participants’ descriptions. Still, the asymmetry of power in the participant-interviewer relationship and its potential impact on data must be considered. Furthermore, persons declining participation and persons who were excluded may experience distress not reflected in the findings as well as not experiencing the distress reported herein. For example, persons already receiving psychotherapy as well as persons not wanting psychotherapy may experience distress of a more severe, shameful, complex and/or debilitating character, e.g., suicidal ideation, which may not have been fully captured in our study. Then again, survivors not reporting a need for psychological support may be less distressed than study participants. The transferability of findings must thus be considered with respect to the sample and its characteristics. Also, many of the concerns (e.g., concerns relating to identity, social inclusion, performance/achievement, and meaning) and manifestations of distress (anxiety, low mood) that the survivors described are considered common experiences among young persons without lived experience of cancer. The study design does not provide insight into whether identified aspects of distress would have emerged if young persons not diagnosed with cancer had been interviewed about past and present challenges.

## Conclusions and clinical implications

Young survivors of adolescent cancer describe feeling physically, socially, and mentally marked by the cancer experience. They struggle with powerlessness, insecurity, social disconnection, loneliness, feelings of being unimportant and failures, and difficulties understanding and managing their experiences. These experiences should be addressed in psychological interventions for the population irrespective of which approach or model used to understand survivors’ difficulties. Findings suggest targeting perceived control, intolerance of uncertainty and/or avoidance of aversive experiences, for example behavioural activation or acceptance and commitment therapy may be beneficial. Indeed, given the diverse manifestations and overlap of distress, trans-diagnostic approaches seem promising. Moreover, exploring social anxiety taking into account experiences of social exclusion, cancer stigma, and alienation appear important. Lastly, supporting the exploration of values and self-knowledge may be highly relevant to promote engagement with therapy among young survivors of adolescent cancer.

Study findings contribute to new knowledge by providing a thorough understanding of salient concerns and experiences among young survivors of adolescent cancer reporting a need for psychological support. This knowledge is crucial to developing psychological treatments that are relevant, engaging, and effective for this population.

## Supporting information

S1 Trial Protocol (PDF)
