# Supplementary material for: Exploration of psychological distress experienced by survivors of adolescent cancer reporting a need for psychological support
Source: PLoS One. 2018 Apr 17;13(4):e0195899. doi: 10.1371/journal.pone.0195899 (PMC5903650; doi:10.1371/journal.pone.0195899)
Supplement: S1 Trial Protocol — (PDF) [file pone.0195899.s001.pdf]

Development of psychological treatment for cancer-related suffering experienced by young people diagnosed with cancer during adolescence

Pediatric oncology care has improved dramatically over the past 20 years and the overall survival rate for childhood cancer is approaching 80 % (Robison et al., 2009). Advances in treatments have ensured this development and children and adolescents struck by cancer experience increasing periods of disease-free survival. Although most children and adolescents diagnosed with cancer survive the disease, challenges remain for these persons and their families. A growing body of literature has shed light on the long-term physical and psychological consequences of cancer and it has been shown that childhood cancer survivors are at risk for psychological problems (Bruce, 2006).

Adolescence is a turbulent period characterized by physical, emotional, social and cognitive changes, being diagnosed with cancer during this period involves challenges that may be especially strenuous to cope with (Epelman, 2013). We have followed a cohort diagnosed with cancer during adolescence from shortly after diagnosis (N=61) up to 10 years after diagnosis investigating HRQOL, anxiety, and depression in comparison to a reference group. Results show that the first phase of survivorship is characterized by low vitality and mental health and increased levels of depression, thereafter rapidly increasing levels of vitality and mental health and decreasing levels of anxiety and depression up to four years after diagnosis. Ten years after diagnosis the cancer group reports higher vitality but in other parts comparable HRQOL and psychological distress as the reference group (Ander et al., 2014). The findings support conclusions by others (Eiser, 2000; Lund, Schmiegelow, Rechnitzer, & Johansen, 2011; Phipps et al., 2014) indicating that long-term survivors of cancer during adolescence are doing well compared to controls without a history of cancer. However, at all assessments a substantial subgroup reports clinically relevant distress and the findings suggest increasing levels of anxiety and depression from four up to 10 years after diagnosis.

The main part of the literature on young persons' suffering related to cancer has conceptualized the psychological symptoms that these persons report as PTSS/PTSD and anxiety. In this project we will identify and describe how young people struck by cancer experience their present and past life-situation, what kind of cancer-related suffering they experience and their ideas about the future (Study A). We will also develop and evaluate psychological treatment based on cognitive behavioral therapy (CBT) to help young people handle the cancer-related psychological suffering they experience (Study B). CBT is effective in treating e.g. anxiety, depression, and PTSD (SBU, 2004; SBU, 2005) but, to our knowledge, no study has evaluated the effect of face-to-face CBT on cancer-related psychological suffering experienced by young people struck by cancer during adolescence. We have used the same procedure as we will use in this project when developing internet-based CBT for irritable bowel syndrome (Ljótsson, Andréewich, et al., 2010; Ljótsson, Falk, et al., 2010) and hypochondriasis (Hedman et al., 2010; Hedman et al., 2011) and currently follow the same procedure to develop CBT for parents of children previously treated for cancer (EPN Dnr 2012/440).

Aims

Study A aims to identify and describe cancer-related suffering among young people struck by cancer during adolescence. Study B aims to develop and evaluate a CBT-based treatment

targeting cancer-related psychological suffering experienced by young people struck by cancer during adolescence.

Design

Study A

Study A has an explorative design. Each participant is interviewed twice with unstructured questions, interviews are audio recorded, transcribed verbatim, and analyzed with inductive content analysis.

Study B

Study B has an uncontrolled, within group design in which CBT is given to young persons (15-25 years) struck by cancer during adolescence. Each person receives up to 15 sessions of face-to-face CBT.

Sample

The same persons participate in Study A and B. Persons are eligible if they are registered in the Swedish Childhood Cancer Registry; are 15-25 years at study-start; were diagnosed with cancer during adolescence (13-18 years); were treated at Akademiska Barnsjukhuset, Uppsala or Astrid Lindgrens Barnsjukhus, Stockholm; have completed cancer treatment; and experience cancer-related psychological suffering. Persons are excluded if they suffer from a psychiatric disorder in immediate need of treatment, if they are in psychotherapy or if they have suicidal ideations (based on a clinical assessment combining self-ratings on the Montgomery-Åsberg Depression Rating Scale (MADRS-S) (Svanborg & Åsberg, 1994) and information from the diagnostic interview Mini International Neuropsychiatric Interview (M.I.N.I.) (Sheehan et al., 1998)).

Since we do not know what cancer-related psychological suffering the participants experience and hence not the effect of CBT on this suffering, power calculations are by necessity a crude approximation. Based on effect sizes obtained in meta-analyses of anxiety and depressive disorders (Butler, Chapman, Forman, & Beck, 2006) we estimate an effect size (Cohen's d) of about 0.8 in Study B. For a power of 80 percent to detect a statistically significant difference and allowing for 25 percent dropout or non-completers, around 20 participants are needed in Study B. Hence, around 20 persons will be included in Study A and B. Inclusion will end when 20 persons or more are included and when data saturation is reached in Study A. Our experience from previous (Hedman et al., 2010; Hedman et al., 2011; Ljótsson, Andréewich, et al., 2010; Ljótsson, Falk, et al., 2010) and ongoing studies is that a sample of around 20 persons provides enough information to develop a CBT protocol.

Treatment

The CBT interventions will be based on a clinical behavior analysis (Dougher, 2000) conducted during the assessment phase of the treatment, which normally extends to the first two or three sessions. The behavior analysis guides the treatment and interventions are chosen based on this analysis and the participant's individual needs. Three therapists work with the treatments, two licensed psychologists and one PTP-psychologist. The CBT is administered once a week. Each session lasts approximately 45 minutes and each participant is given 10-15 sessions. If a participant has remaining needs for psychological treatment after these sessions he/she is referred to the regular health care.

Data collection and Procedure

The protocol has been approved by the medical directors at the centres for paediatric oncology in Uppsala and Stockholm and the person in charge of the Swedish Childhood Cancer Registry. See Attachment 12 for a visual presentation of the procedure.

1. Persons 15-25 years diagnosed with cancer during adolescence (13-18 years) who were treated at Akademiska Barnsjukhuset in Uppsala or Astrid Lindgrens Barnsjukhus in Stockholm and are registered in the Swedish Childhood Cancer Registry are identified via this registry. Data about personal identity number; name; gender; diagnosis and date of diagnosis are collected via the registry by our research group with assistance from register personnel. At the same occasion addresses to potential participants are identified via Statens Personadressregister (SPAR). Data collected via the two registries are saved on a USB flash drive and brought to the principal investigator (professor Louise von Essen).
2. Telephone numbers to potential participants 18-25 years are identified via internet search engines.
3. Telephone numbers to and information about treatment status (on/off) of potential participants 15-17 years are identified by medical personnel at the pediatric oncology units at Akademiska Barnsjukhuset in Uppsala or Astrid Lindgrens Barnsjukhus in Stockholm.
4. Persons are contacted via telephone, in alphabetical order, by a psychologist and receive short information about the study. Thereafter potential participants are asked whether they have the possibility to participate in Study A and Study B in Uppsala or Stockholm respectively. If so additional information is provided about the aims and procedure of the project and about CBT. Potential participants are thereafter asked whether they experience cancer-related psychological suffering and whether they participate in psychological treatment. Persons ≥ 18 years are asked if they have finished cancer treatment. Those who no longer are on cancer treatment, do not participate in psychological treatment and experience cancer-related suffering are offered an assessment meeting with a psychologist and are sent written information about the project (Attachment 4.1).
5. If inclusion has not finished (around 20 have not been included and data saturation has not been reached) when all persons with identified telephone numbers have been contacted, persons for whom telephone numbers cannot be identified are sent written information about the study (Attachment 4.2) in alphabetical order. The number contacted depends on the number of persons included via telephone and the inclusion rate via telephone. Persons who are interested in participating in the study are directed to a secure website where they can register name and contact information. Persons who have registered at the website are contacted via telephone by a psychologist and receive more information. Potential participants are asked whether they experience psychological suffering connected to the cancer and whether they participate in psychological treatment. Persons ≥ 18 years are asked if they have finished cancer treatment. Those who no longer are on treatment, do not participate in psychological treatment and experience cancer-related suffering are offered an assessment meeting with a psychologist.
6. Caregivers of persons 15-17 years who are booked for an assessment meeting are informed about the study via telephone and via written information (Attachment 4.4).

7. During the assessment meeting participants are first asked to complete the MADRS-S (Attachment 5.1) and thereafter the M.I.N.I. structured diagnostic psychiatric interview for DSM-IV and ICD-10 (MINI) (Sheehan et al., 1998) to assess psychiatric disorders is administered to identify persons with psychiatric problems in immediate need of treatment. If a person's scores on one or both of these assessments indicate a risk for suicide a supervisor (licensed psychologist) working in the project calls the person to make a final judgment. Participation is thereafter offered to persons who fulfill the inclusion criteria and are not excluded. In connection to this the psychologist performs an assessment of whether persons 15-17 years understand what participation implies in order to judge whether their consent to participate in the study is sufficient. If a person does not seem to understand the implication of participation his/her caregiver/s receive information (Attachment 4.5) and is/are asked to sign consent form (Attachment 4.6) which the potential participants is asked to bring to the psychologist at the first interview session. The potential participant and his/her caregiver/s are informed that participation cannot start until caregiver/s' consent/s have been collected. Potential participants are once again provided written information (Attachment 4.1 or Attachment 4.2), and asked for written consent (Attachment 4.3.1 when caregiver consent is collected, Attachment 4.3.2 for others). After consent is provided persons are asked to complete the self-assessment forms (Attachments 5.2-5.13), see below.

   The Beck Anxiety Inventory (BAI): The BAI (Beck, Epstein, Brown, & Steer, 1988) consists of 21 items measuring symptoms of anxiety (Attachment 5.2).
   The PTSD Checklist - Civilian (PCL-C): The PCL-C (Weathers, Litz, Herman, Huska, & Keane, 1993) consists of 17 items measuring symptoms of PTSD as defined in the B (re-experiencing), C (avoidance), and D (hyperactivity) criteria in DSM-IV (American Psychiatric Association, 2000). In this study the items are keyed to the person's cancer disease (Attachment 5.3).
   Fatigue Assessment Scale (FAS): FAS (Michielsen, De Vries, & Van Heck, 2003) is a 10-item fatigue scale. The person is asked to answer how he/she usually feels on a 5-point rating scale that varies from 1, never, to 5, always (Attachment 5.4).
   Sheehan Disability Scale (SDS): The SDS (Sheehan, 1983) assesses functional impairment in three domains; work/school, social and family life. The participant rates the extent to which these three domains are impaired by his or her symptoms on a 10 point visual analog scale (Attachment 5.5).
   Short Health Anxiety Inventory (SHAI): The SHAI (Salkovskis, Rimes, Warwick, & Clark, 2002) consists of 18 items most highly correlated with the Health Anxiety Inventory (64 items) measuring health anxiety. Each item consists of four statements and the respondent is asked to select the statement that best reflects his/her experience the last week (Attachment 5.6).
   Body Image Scale (BIS): Body Image Scale (BIS): BIS (Hopwood, Fletcher, Lee, & Al Ghazal, 2001) is a 10-item scale developed for use in clinical trials to assess body image changes in cancer patients (Attachment 5.7).
   The Penn State Worry Questionnaire (PSWQ): The PSWQ (Meyer, Miller, Metzger, & Borkovec, 1990) consists of 16 items measuring excessive worry (Attachment 5.8).

Rumination Scale of the Response Style Questionnaire (RSQ): The rumination scale of RSQ (Nolen-Hoeksema, 1991) consists of 22 statements and measures rumination as a response to symptoms of depression (Attachment 5.9).
Patient Health Questionnaire-15 (PHQ-15): The PHQ-15 (Kroenke, Spitzer, & Williams, 2002) measures severity of somatic symptoms and comprises 15 somatic symptoms that are scored from 0 (“not bothered at all”) to 2 (“bothered a lot”) (Attachment 5.10).
Acceptance and Action Questionnaire (AAQ-II): AAQ-II (Bond et al., 2011) consists of 10 items measuring experiential avoidance (Attachment 5.11).
Questions about demographic data and the disease (Attachment 5.12 and 5.13).

If caregiver/s does/do not consent participation is not continued.

Study A
Each participant is interviewed twice and is asked to describe cancer-related suffering in terms of thoughts and concerns about the past, present, and future related to the cancer disease. The interview starts with the question: “Could you please tell me about your thoughts and concerns related to your disease?” Follow-up questions are posed according to the respondent’s answers. The interviews are conducted by one of the psychologists who work with the treatment in Study B. The interviews are audio-recorded and transcribed verbatim.

Study B
Data is collected shortly after and 3 months after end of treatment. Participants answer questions in the questionnaires 5.1-5.11 at these assessments and MINI is administered at the same assessments by a psychologist (not the treating psychologist).

Data analyses
Study A
The audio-recorded interviews are transcribed verbatim. A computer program is used to support and facilitate the inductive thematic analysis of the interview material. Analysis is done in parallel to data collection in order to observe when data saturation is reached. Recording units are structured in categories, and the analysis continues until no new categories emerge.

Study B
The treatment effect is evaluated by estimates of effect size, reliable change indices, and the proportion of individuals achieving clinically significant change.

ETHICAL AND METHODOLOGICAL CONSIDERATIONS AND IMPORTANCE

Participation is voluntary and it is possible to withdraw without any consequences at any time. The participants’ confidentiality is guaranteed and consideration is taken to their integrity, dignity, and vulnerability. All data are handled according to *Personuppgiftslagen* (PUL 1998:204) and *Patientdatalagen* (208:355). The informed consent ensures that participants are well aware of the conditions of participation.

Lagen om etikprövning (Lag 2003:460) 18 § states that persons who are 15-17 years and who realize the implications of participation in research should be informed and provide consent (as stated in 16 and 17 §§ in *Lagen om etikprövning* (Lag 2003:460)). In order to overcome the risk that persons younger than 18 years provide consent without understanding the

implications of participation an assessment of their understanding of what participation implies will be made by a psychologist. If a person does not demonstrate sufficient understanding of the implications of participation caregivers are contacted and asked for written consent.

Using an uncontrolled design may be an ethical concern since persons' efforts will yield data of less scientific value than a controlled design. However, given the paucity of knowledge about the cancer-related psychological suffering of this group and the fact that we know of no other study of individual face-to-face CBT for this group, we believe that it is justified to first perform a pilot study to estimate the effectiveness of the intervention

To date there is no evidence-based psychological treatment to offer young people struck by cancer during adolescence. An overarching aim with Study A and B is to gain knowledge that can form the basis for a treatment program that can be evaluated in a controlled study. To develop such a treatment the long-term consequences of being diagnosed with cancer during adolescence needs to be more fully identified, which is the aim of Study A. Study B is the first step in the process of developing a treatment for young survivors of adolescent cancer. Both studies will make important contributions in the process of generating psychological treatment that can be offered to young people suffering from the distressing experiences related to having had cancer during adolescence.

References

American Psychiatric Association. (2000). *Diagnostic and statistical manual of mental disorders* (4th ed.). Washington, DC: American Psychiatric Association.

Ander, M., Grönqvist, H., Cernvall, M., Engvall, G., Hedström, M., Ljungman, G., … von Essen, L. (2014). *Health-related quality of life, anxiety, and depression after cancer during adolescence in comparison to a healthy reference group: A 10-year follow up.* Manuscript in preparation.

Beck, A. T., Epstein, N., Brown, G., & Steer, R. A. (1988). An inventory for measuring clinical anxiety: Psychometric properties. *Journal of Consulting and Clinical Psychology, 56*(6), 893–897.

Bond, F. W., Hayes, S. C., Baer, R. A., Carpenter, K. M., Guenole, N., Orcutt, H. K., … Zettle, R. D. (2011). Preliminary psychometric properties of the Acceptance and Action Questionnaire–II: A revised measure of psychological inflexibility and experiential avoidance. *Behavior Therapy, 42*(4), 676–688. doi: 10.1016/j.beth.2011.03.007

Bruce, M. (2006). A systematic and conceptual review of posttraumatic stress in childhood cancer survivors and their parents. *Clinical Psychology Review, 26*(3), 233–256. doi:10.1016/j.cpr.2005.10.002

Butler, A. C., Chapman, J. E., Forman, E. M., & Beck, A. T. (2006). The empirical status of cognitive-behavioral therapy: A review of meta-analyses. *Clinical Psychology Review, 26*(1), 17–31. doi:10.1016/j.cpr.2005.07.003

Dougher, M. J. (2000). *Clinical behavior analysis.* Reno: Context press.

Eiser, C. (2000). Examining the psychological consequences of surviving childhood cancer: Systematic review as a research method in pediatric psychology. *Journal of Pediatric Psychology, 25*(6), 449–460. doi:10.1093/jpepsy/25.6.449

Epelman, C. L. (2013). The adolescent and young adult with cancer: State of the art – psychosocial aspects. *Current Oncology Reports*, *15*(4), 325–331. doi:10.1007/s11912-013-0324-6

Hedman, E., Andersson, G., Andersson, E., Ljótsson, B., Rück, C., Asmundson, G. J. G., & Lindefors, N. (2011). Internet-based cognitive-behavioural therapy for severe health

anxiety: Randomised controlled trial. *The British Journal of Psychiatry, 198*(3), 230–236. doi:10.1192/bjp.bp.110.086843

Hedman, E., Ljótsson, B., Andersson, E., Rück, C., Andersson, G., & Lindefors, N. (2010). Effectiveness and cost offset analysis of group CBT for hypochondriasis delivered in a psychiatric setting: An open trial. *Cognitive Behaviour Therapy*, *39*(4), 239–250. doi:10.1080/16506073.2010.496460

Hopwood, P., Fletcher, I., Lee, A, & Al Ghazal, S. (2001). A body image scale for use with cancer patients. *European Journal of Cancer, 37*(2), 189–197.

Kroenke, K., Spitzer, R. L., & Williams, J. B. W. (2002). The PHQ-15: Validity of a new measure for evaluating severity of somatic symptoms. *Psychosomatic Medicine, 64*(2), 258–266.

Ljótsson, B., Andréewitch, S., Hedman, E., Rück, C., Andersson, G., & Lindefors, N. (2010). Exposure and mindfulness based therapy for irritable bowel syndrome – An open pilot study. *Journal of Behavior Therapy and Experimental Psychiatry*, *41*(3), 185–190. doi:10.1016/j.jbtep.2010.01.001

Ljótsson, B., Falk, L., Vesterlund, A. W., Hedman, E., Lindfors, P., Rück, C., … Andersson, G. (2010). Internet-delivered exposure and mindfulness based therapy for irritable bowel syndrome – A randomized controlled trial. *Behaviour Research and Therapy, 48*(6), 531–539. doi:10.1016/j.brat.2010.03.003

Lund, L. W., Schmiegelow, K., Rechnitzer, C., & Johansen, C. (2011). A systematic review of studies on psychosocial late effects of childhood cancer: Structures of society and methodological pitfalls may challenge the conclusions. *Pediatric Blood & Cancer, 56*(4), 532–543. doi:10.1002/pbc.22883

Meyer, T. J., Miller, M. L., Metzger, R. L., & Borkovec, T. D. (1990). Development and validation of the Penn State Worry Questionnaire. *Behaviour Research and Therapy, 28*(6), 487–495. doi:10.1016/0005-7967(90)90135-6

Michielsen, H. J., De Vries, J., & Van Heck, G. L. (2003). Psychometric qualities of a brief self-rated fatigue measure. *Journal of Psychosomatic Research*, *54*(4), 345–352. doi:10.1016/S0022-3999(02)00392-6

Nolen-Hoeksema, S. (1991). Responses to depression and their effects on the duration of depressive episodes. *Journal of Abnormal Psychology*, *100*(4), 569–582. doi:10.1037/0021-843X.100.4.569

Phipps, S., Klosky, J. L., Long, A., Hudson, M. M., Huang, Q., Zhang, H., & Noll, R. B. (2014). Posttraumatic stress and psychological growth in children with cancer: Has the traumatic impact of cancer been overestimated? *Journal of Clinical Oncology*, *32*(7), 641–646. doi:10.1200/JCO.2013.49.8212

Robison, L. L., Armstrong, G. T., Boice, J. D., Chow, E. J., Davies, S. M., Donaldson, S. S., … Zeltzer, L. K. (2009). The Childhood Cancer Survivor Study: A National Cancer Institute-supported resource for outcome and intervention research. *Journal of Clinical Oncology*, *27*(14), 2308–2318. doi:10.1200/JCO.2009.22.3339

Salkovskis, P. M., Rimes, K. A., Warwick, H. M. C., & Clark, D. M. (2002). The Health Anxiety Inventory: Development and validation of scales for the measurement of health anxiety and hypochondriasis. *Psychological Medicine*, *32*(5), 843–853. doi:10.1017}S0033291702005822

SBU - Statens beredning för medicinsk utvärdering. (2004). *Behandling av depressionssjukdomar*. *En systematisk litteraturöversikt* (SBU Publication No. 166). Stockholm: Statens beredning för medicinsk utvärdering.

SBU - Statens beredning för medicinsk utvärdering. (2005). *Behandling av ångestsyndrom. En systematisk litteraturöversikt* (SBU Publication No. 171). Stockholm: Statens beredning för medicinsk utvärdering.

Sheehan, D. V. (1983). *The Anxiety Disease.* New York: Charles Scribner & Sons.

Sheehan, D. V., Lecrubier, Y., Sheehan, K. H., Amorim, P., Janavs, J., & Weiller, E. (1998). The Mini-International Neuropsychiatric Interview (M.I.N.I.): The development and validation of a structured diagnostic psychiatric interview for DSM-IV and ICD-10. *Journal of Clinical Psychiatry*, *59*(20), 22–33.

Svanborg, P., & Åsberg, M. (1994). A new self-rating scale for depression and anxiety states based on the Comprehensive Psychopathological Rating Scale. *Acta Psychiatrica Scandinavica*, *89*(1), 21–28. doi:10.1111/j.1600-0447.1994.tb01480.x

Weathers, F. W., Litz, B. T., Herman, D. S., Huska, J. A., & Keane, T. M. (1993). *The PTSD Checklist (PCL): Reliability, validity, and diagnostic utility*. Paper presented at the Annual Conference of the International Society for Traumatic Stress Studies, San Antonio, TX.
